# Identification of genes with high heterogeneity of expression as a predictor of different prognosis and therapeutic responses in colorectal cancer: a challenge and a strategy

**DOI:** 10.1186/s12935-022-02694-9

**Published:** 2022-09-05

**Authors:** Ebrahim Salehitabar, Mohammad Mahdevar, Ali Valipour Motlagh, Farzad Seyed Forootan, Sara Feizbakhshan, Dina Zohrabi, Maryam Peymani

**Affiliations:** 1 Department of Biology, Faculty of Science, NourDanesh Institute of Higher Education, Isfahan, Iran; 2 Cellular, Molecular and Genetics Research Center, Isfahan University of Medical Sciences, Isfahan, Iran; 3 Medical Genetics Research Center of Genome, Isfahan University of Medical Sciences, Isfahan, Iran; 4 Department of Biology, Faculty of Basic Sciences, Shahrekord Branch, Islamic Azad University, Shahrekord, Iran; 5 Legal Medicine Research Center, Legal Medicine Organization, Tehran, Iran

**Keywords:** High heterogeneity, Tumor sample, Mutation, Gene expression, Cancer treatment

## Abstract

**Background:**

Molecular heterogeneity is one of the most important concerns in colorectal cancer (CRC), which results in a wide range of therapy responses and patient prognosis. We aimed to identify the genes with high heterogeneity of expression (HHE) and their relation with prognosis and drug resistance.

**Methods:**

Two cohort studies, the cancer genome atlas (TCGA) and the GSE39582, were used to discover oncogenes genes with HHE. The relationship between identified genes with clinical and genomic characteristics was evaluated based on TCGA data. Also, the GDSC and CCLE data were used for drug resistance and sensitivity. Sixty CRC samples were used to validate the obtained data by RT-qPCR.

**Results:**

Findings revealed that 132 genes with HHE were found to be up-regulated in both cohorts and were enriched in pathways such as hypoxia, angiogenesis, and metastasis. Forty-nine of selected genes related to clinical and genomic variables, including stage, common mutations, the tumor site, and microsatellite state that were ignored. The expression level of *CXCL1*, *SFTA2*, *SELE*, and *SACS* as genes with HHE were predicted survival patients, and RT-qPCR results demonstrated that levels of *SELE* and *SACS* had HHE in CRC samples. The expression of many identified genes like *BGN*, *MMP7*, *COL11A1*, *FAP*, *KLK10*, and *TNFRSE11B* was associated with resistance to chemotherapy drugs.

**Conclusions:**

Some genes expression, including *SELE, SACS, BGN, KLK10, COL11A1, and TNFRSE11B* have an oncogenic function with HHE, and their expression can be used as indicators for differing treatment responses and survival rates in CRC.

**Supplementary Information:**

The online version contains supplementary material available at 10.1186/s12935-022-02694-9.

## Introduction

Colorectal cancer (CRC) is one of the most common types of cancer in the world with a high mortality rate [1]. Based on the genetic standpoint, CRC is a highly heterogeneous and multifactorial disease, so one of the most effective treatments for this disease is targeted therapy based on the individual molecular characteristics of the patient, and personalized medicine [2]. Therefore, identifying the molecular properties associated with the disease and classifying them can provide appropriate drugs.

Variable therapeutic responses and different survival rates for patients with the same symptoms and clinical features have been an important challenge in CRC. One of the main reasons for the mentioned feature is the existence of different molecular patterns among the samples with the same clinical features, which is known as heterogeneity [3]. In general, heterogeneity can be assessed at four levels, including gene expression, mutations, epigenetics, and microenvironment [4]. Therefore, many studies have classified cancer samples according to the mentioned characteristics, the most important of which has been the classification based on genomic features such as methylation status of CpGs, mutation, and microsatellite instability (MSI) [5, 6]. Studies have shown that there is a high rate of mutations in a large number of genes among CRC samples, the most common of which are mutations in the *APC*, *TP53*, and *KRAS* genes [7]. Furthermore, CRC epigenetic studies have shown that changes in epigenetic markers in cancer samples compared to normal occur significantly in many genes, and these markers differ from one cancer sample to another [8]. These results clearly indicate that molecular characteristics in CRC specimens can be very variable and cause different phenotypes.

Gene expression is one of the main variables with the high degree of heterogeneity in some cancers, especially in CRC [9]. Moreover, previous studies have reported that changes in gene expression are associated with prognosis and drug resistance and increase the risk of malignancy in many cancers [10, 11]. Therefore, genes with high heterogeneity of expression (HHE) can cause different therapeutic responses and different prognosis in CRC patients. To date, most heterogeneous studies in CRC have been done more on genomic features and the identification of genes with HHE has been less identified. In this study, using transcriptomic data of two CRC cohorts, the genes with HHE were assessed. Following that, in silico analysis was used to investigate the role of identified genes in patient prognosis and their relationship with drug resistance. In addition, the expression of two candidate genes was examined in CRC samples by the RT-qPCR method.

## Material and methods

### Data collection

Transcriptome data from two CRC cohort studies, the cancer genome atlas (TCGA) and the GSE39582 dataset, were used in this work. CRC raw data (RNA-Seq) and clinical dataset from TCGA database (https://portal.gdc.cancer.gov/) were downloaded by R “TCGAbiolinks” package [12]. These data included 480 tumor samples at different stages and 41 normal samples. By utilizing “edgeR” package and by considering CPM criterion (CPM less than 10 in 70% of samples), genes with low expression and close to zero were removed from raw counts data. Then the data was normalized by TMM method and was transferred into log2 [13]. The resulting matrix was used for all analyzes used in this study. The GSE39582 raw data files (.CEL format), performed on an Affymetrix Human Genome U133 Plus 2.0 Array were obtained directly from the Gene Expression Omnibus (GEO) database and R “limma” package and RMA method were applied to background correction, normalization, and log2 transformation [14]. This dataset includes 566 tumor samples at different stages and 19 normal samples. In addition, using the “arrayQualityMetrics” package, the quality of each presentation was evaluated and samples that lacked quality were excluded from the study. Finally, for several probes related to one gene, the mean value was considered and the resulting expression matrix was used for the study analysis process.

### Enrichment and survival analysis

To enrich and identify the signature pathways for genes identified with HHE, MSigDB data from the Enrichr database (https://maayanlab.cloud/Enrichr) was employed. Clinical data from the TCGA database were used to survival analysis and to assess the relationship between the expression level of genes and the patient's prognostic. In this regard, patients whose number of survival days were zero, one and NA were excluded from the study. Also, for cases in which patients died, only those who had a tumor at the time of death were considered. To assess the correlation between gene expression with patient survival, the expression of each gene in the normalized expression matrix was calculated as the Z-score. Finally, Z-score data and expression of candidate genes were used for survival analyzes. The risk score was calculated with the following formula: risk score = β1*Exp1 + β2*Exp2 + βi*Expi, where β indicates the multivariate coefficient value and Exp screens the gene expression level.

### Analysis of genomic data

DNAseq data available for each sample in the TCGA database were used to identify common mutations as well as the effect of mutations on gene expression. For this purpose, MAF data of all CRC samples were downloaded with pipeline Mutect2 [15], and then the frequency and type of mutations in all samples were evaluated by R “maftools” package [16]. To evaluate the effect of microsatellite status on candidate gene expression, using data from the TCGA database, the samples were divided into two groups: microsatellite instability (MSI) and microsatellite stable (MSS). According to the obtained information, 127 samples had MSS status and 40 samples had MSI status. Finally, differential expression analysis was performed for candidate genes in MSI and MSS groups.

### Drug resistance and sensitivity

The cancer cell line encyclopedia (CCLE) and genomics of drug sensitivity in cancer (GDSC) databases were utilized to investigate the relationship between the expression of genes with HHE and drug resistance. In this regard, using R “pharmachoGX” package, expression information for candidate genes and IC50 of different drugs were extracted and the relationship between the expression of candidate genes and the IC50 level for each drug was calculated through the Pearson correlation test.

### Sample collection, cDNA synthesis and RT-qPCR

Thirty CRC samples with 30 adjacent normal tissues were obtained from the tumor bank of Iran, and all samples were taken with the volunteers' agreement from patients. This study was approved by the Biomedical Ethics Committee of the Islamic Azad University with the Ethics Code of IR.IAU.TNB.REC.1400.005. Before use, cancer samples were approved by a pathologist and kept in liquid nitrogen. All tissues were washed with PBS- three times before RNA extraction to remove necrotic cells. RNA extraction was performed by TRIzol reagent (Invitrogen) according to the manufacturer's instructions and DNase treatment was performed to eliminate possible DNA contamination (Fermentas). The synthesis of cDNA was carried out according to the manufacturer's instructions using a Yekta Tajheiz kit.. Cellular RNA expression levels were measured using specific primers including *SELE* (forward: 5′-GATGTTGAATGCCCACAGGC-3′ and reverse: 5′-GTAACCCTCGCACAGAGCAT-3′), *SACS* (forward: 5′-GTGCGCGATGTGAAGGAAC-3′ and reverse: 5′-CAAATCGACCTCGCGGC-3′), and SYBR PrimeScript RT-PCR (TaKaRa). The relative expression of each gene was normalized with the amount of B-actin as an internal control. The expression of each gene in each sample was calculated based on 2^−△Ct^.

### Statistical analysis

The R programming language (v 3.2) was used to analyze and preprocess RNA-seq and microarray data, with the latest package updates supplied in this study. To perform the differential expression analysis, linear model method was utilized and false discovery rate (FDR) was considered for a significant level. GraphPad prism (v 8.4) software was used to display and draw diagrams. Cox regression test was used to analyze the association of gene expression with patients' prognosis and longRank level < 0.01 was considered as a statistical significance. Cytoscape software (v 4.1) was used to draw and categorize the obtained data.

## Results

### Identification of 132 genes in CRC as candidate genes with HHE

**Fig. 1:**
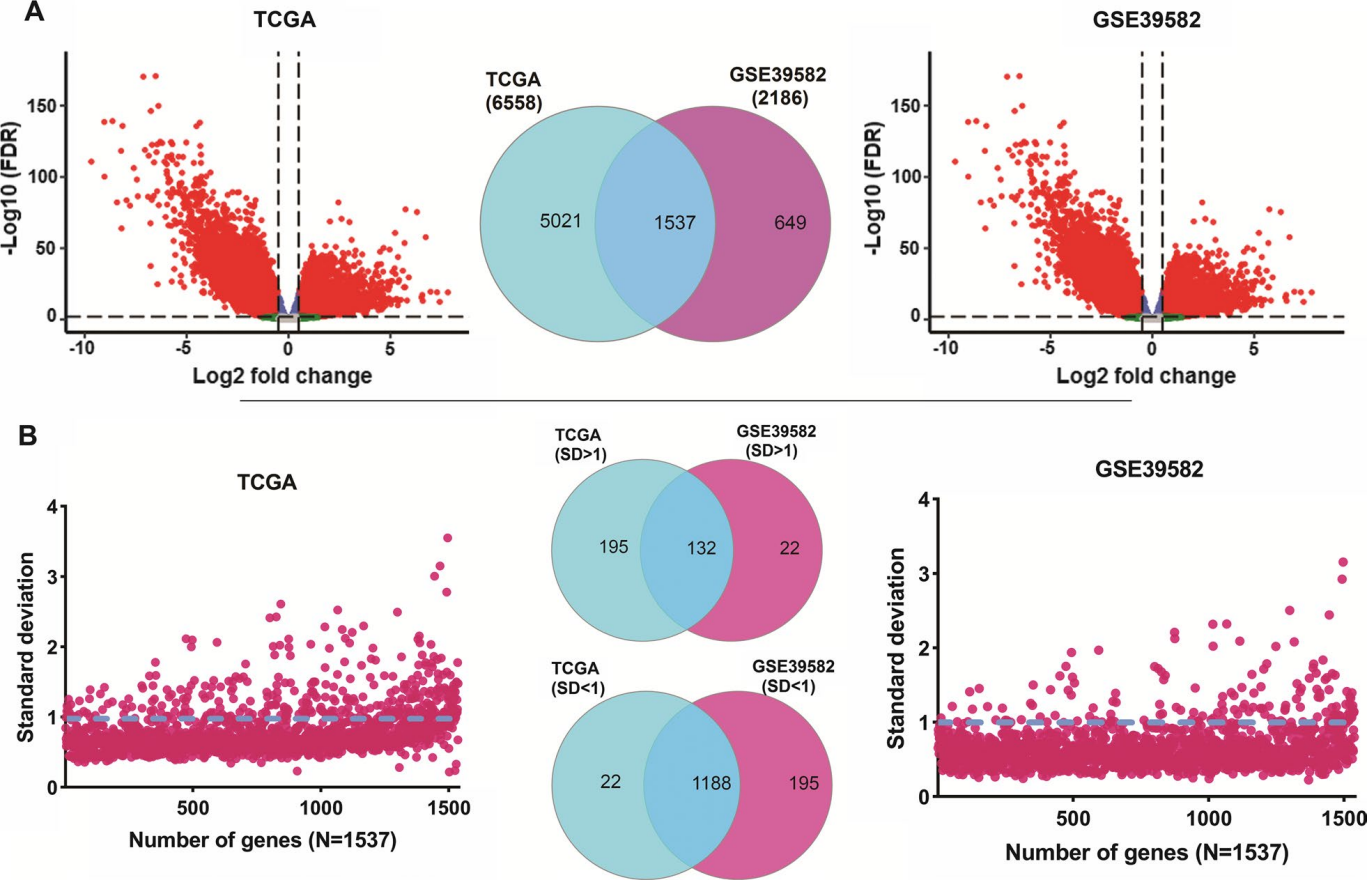
Association of 132 high heterogeneous oncogenic genes in CRC. **A** Volcano plot for differential expression of CRC double cohorts including TCGA and GSE39582 data as well as similar two cohort genes via venn digram. The linear model test was used to analyze the differential expression and logFC > 0.5 and FDR < 0.01 were considered. **B** The standard division scatter plot for the common increase genes was shown in their two common and non-common cohorts. Genes with more than 1 SD in both cohorts were selected (FDR, false discover rate; SD, standard deviation)

To identify genes with HHE, two cohort studies (GSE39582 and TCGA) were used for this project. Examining the quality of GSE39582 samples revealed that 24 of the 585 samples had low quality and were significantly different from other samples, therefore they were eliminated from the analysis. In the first stage, the differential expression of all genes in cancer samples was evaluated in comparison with normal samples, and genes that had oncogenic potential were considered for continuing analysis. Our results showed that under the threshold of FDR < 0.01 and | log FC | > 0.5, 1537 similar up-regulated genes were identified in two cohorts (Fig. 1A). Following that, the expression distributions of 1537 genes identified in both cohort studies were determined using the standard deviation calculation among cancer samples. The results showed that 132 genes in both studies had highly variable expression (SD > 1) between samples and were selected as candidate genes to continue the analysis (Fig. 1B and Additional file 1: Table S1). In addition, 1188 genes had less expression changes between cancer samples (Fig. 1B). These findings imply that 132 genes in CRC could be good candidates for HHE genes and that they may play a role in the discrepancies in therapeutic responses and survival.

### Heterogeneous genes activity in angiogenesis, metastasis, inflammation, and related mutation pathways

**Fig. 2:**
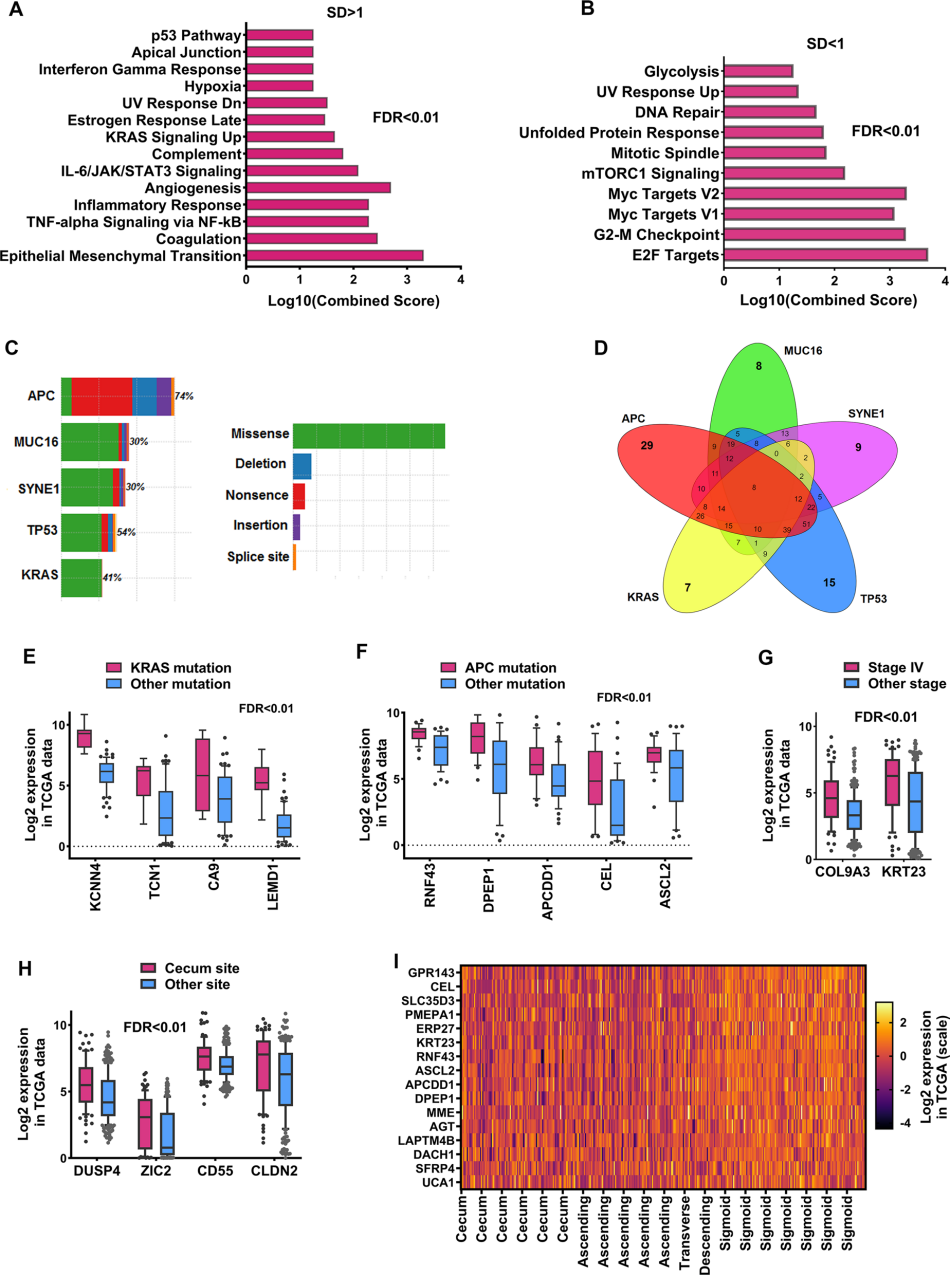
Enrichment of high and low heterogeneity genes as well as the relationship of high heterogeneity genes to clinical and genomic features. **A** Pathways related to identified genes with HHE based on MsigDB data. **B** The pathways related to genes with low heterogeneity of expression level between two cohorts TCGA and GSE39582 based on MsigDB data with FDR < 0.01. **C**, **D** Common mutations based on DNA-seq data and the type of mutation were shown in CRC samples. Venn diagram was used to identify a sample that has only one of the most common mutations including *APC*, *KRAS*, *TP53*, *MUC16,* and *SYNE1*. (E-I) Correlation of 132 identified genes with high heterogeneity of expression level with clinical and genomic characteristics in CRC samples based on the TCGA data. Linear model test was used to assess the differential expression and FDR < 0.01 was considered as statistical significance

In order to gain insight into the pathways involved for genes with the high and the low degree of heterogeneity between CRC specimens, candidate genes were enriched using the MSigDB database. The results showed that the pathways associated with high heterogeneity, were more closely related to the pathways of angiogenesis, metastasis, inflammation, and mutation-related pathways (Fig. 2A, FRD < 0.01). Moreover, genes with low heterogeneity were associated with pathways that relate to the main characteristics of cancer cells such as cell division and glycolysis (Fig. 2B, FDR < 0.01). These results indicated that the most important pathways that could be different from one sample to another sample in CRC are more related to inflammatory pathways, angiogenesis, metastasis, hypoxia, and mutation-related pathways. As a result, these pathways, together with the main cancer pathways, could be good targets for personalized medicine and focused treatment.

### Some HHE genes have a link to the sample's clinical and genetic features

**Fig. 3:**
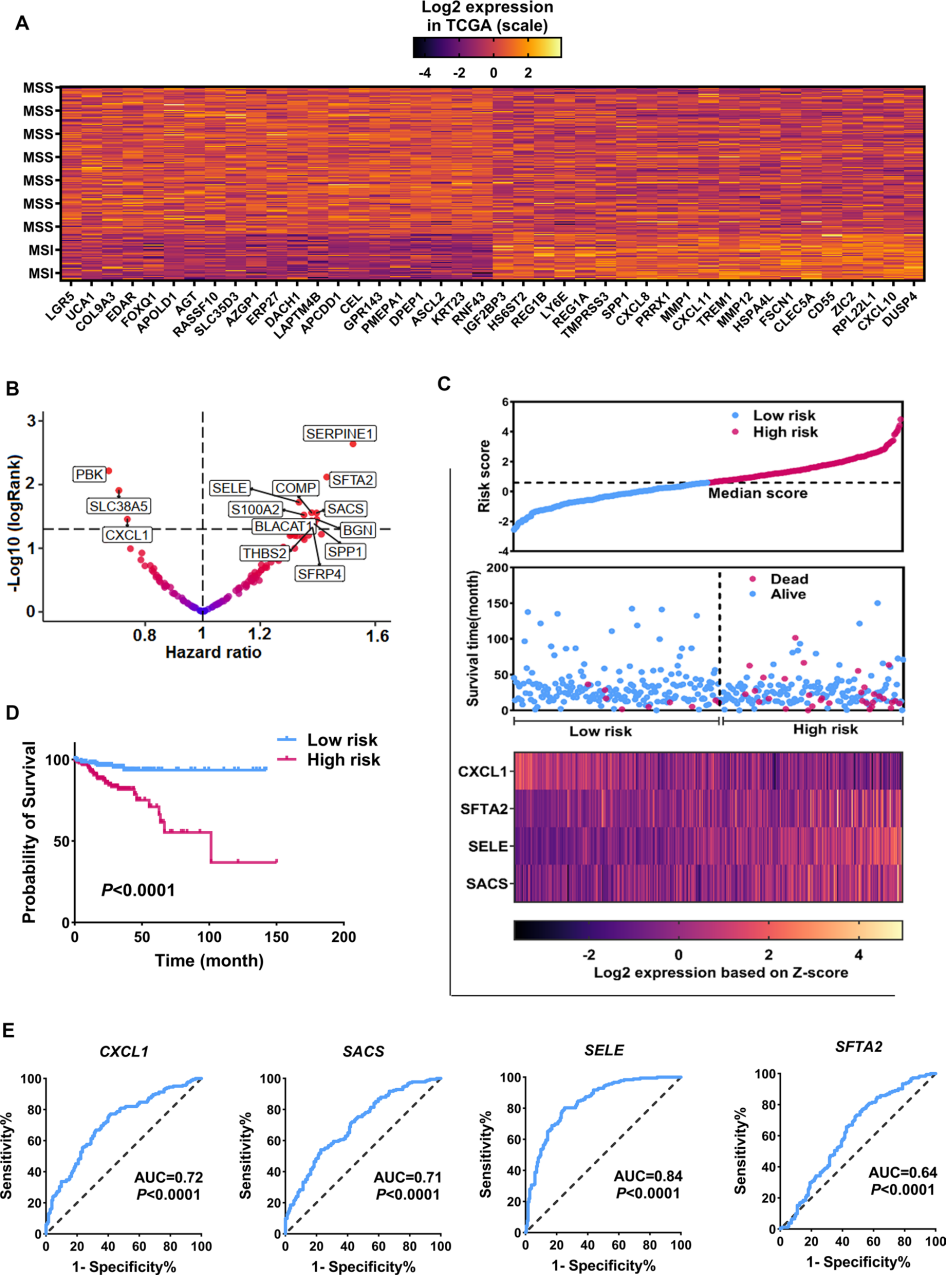
Association of *CXCL1*, *SFTA2*, *SELE* and *SACS* expression with prognosis of CRC patients. **A** Differential expression for identified genes in MIS compared to MSS based on TCGA data. **B** Volcano plot for univariate Cox regression results for genes with HHE and HR > 1 and logRank < 0.01 were considered for selection of genes related to patients survival. **C** Calculate patients' risk score for four genes *CXCL1*, *SFTA2*, *SELE*, and *SACS* using their expression level and beta coefficient of multivariate Cox regression test. The calculated median score was used to divide patients into high-risk and low-risk groups. **D** Kaplan Meier is shown for high risk patients versus low risk patients. **E** Sensitivity and specificity of expression level of *CXCL1*, *SFTA2*, *SELE,* and *SACS* from high risk group against low risk

Since oncologists consider genomic and clinical features in the treatment and prognosis of CRC, we were looking for genes whose expression was not affected by these factors and specialists pay less attention to them. Thus, using TCGA data, the expression level of HHE genes was compared to clinical parameters including stage pathology, sample location in the colon region, age, gender, and BMI (body mass index) as well as genomic aspects such as mutation and genome instability (MSI and MSS). The first step was to acquire data on CRC mutations and determine their frequency. The outcomes displayed that five genes including *APC*, *KRAS*, *TP53*, *MUC16*, and *SYNE1* had the most frequency among other mutant genes (Fig. 2C). Since some samples could have two or more mutations at the same time, to evaluate the impact of the mutation on the expression level of candidate genes, the cancer samples were collected in a way that have only one type of mentioned mutations (Fig. 2D). The results indicated that the expression of five HHE genes was richer in groups with *KRAS* mutations, and the expression of four HHE genes was considerably greater in the group with an *APC* gene mutation compared to other groups (Fig. 2E and F, FDR < 0.01). Moreover, none of the candidate genes in the mutated groups in *MUC16* and *SYNE1* genes were significant. In addition, the evaluation results of the associated HHE genes with stage showed that only two genes including *COL9A3*, and *KRT23* were significantly associated with stage IV (Fig. 2G, FDR < 0.01). Analyzes of the relationship between HHE genes and the tumor site in the colon region indicated that four genes, including *DUSP4*, *ZIC2*, *CD55*, and *CLDN2* significantly increased in the cecum region (Fig. 2H, FDR < 0.01). In addition, 16 genes in the sigmoid region were more expressed compared to other areas of the colon, and no significant genes were identified for other genes and other areas of the colon (Fig. 2I, FDR < 0.01). The relationship between candidate gene expression and microsatellite status was evaluated and the TCGA samples were divided into two groups according to mentioned features: microsatellite instability (MSI) and microsatellite stable (MSS). The expression of many HHE genes were associated with MSI and MSS status so that 21 genes of HHE increased in samples with MSS status compared to MIS (Fig. 3A, FDR < 0.05). In contrast, the expression of 21 genes in the MIS group increased compared to the expression of MSS (Fig. 3A, FDR < 0.05). Finally, the relationship between the expression of HHE genes and clinical features of patients including, age, gender, and BMI were evaluated, and none of the candidate genes were associated with the mentioned characteristics. These results indicate that the expression level of some HHE genes can be related to clinical and genomic features, and the observed different expression behaviors in them associated with these features. Finally, 49 HHE genes were associated with clinical and genomic features that were removed from the list of candidate genes for further analysis. All results are briefly summarized in Additional file 2: Table S2.

### Association of gene expression with HHE including *CXCL1*, *SFTA2*, *SELE*, and *SACS* with the survival of CRC patients

**Fig. 4:**
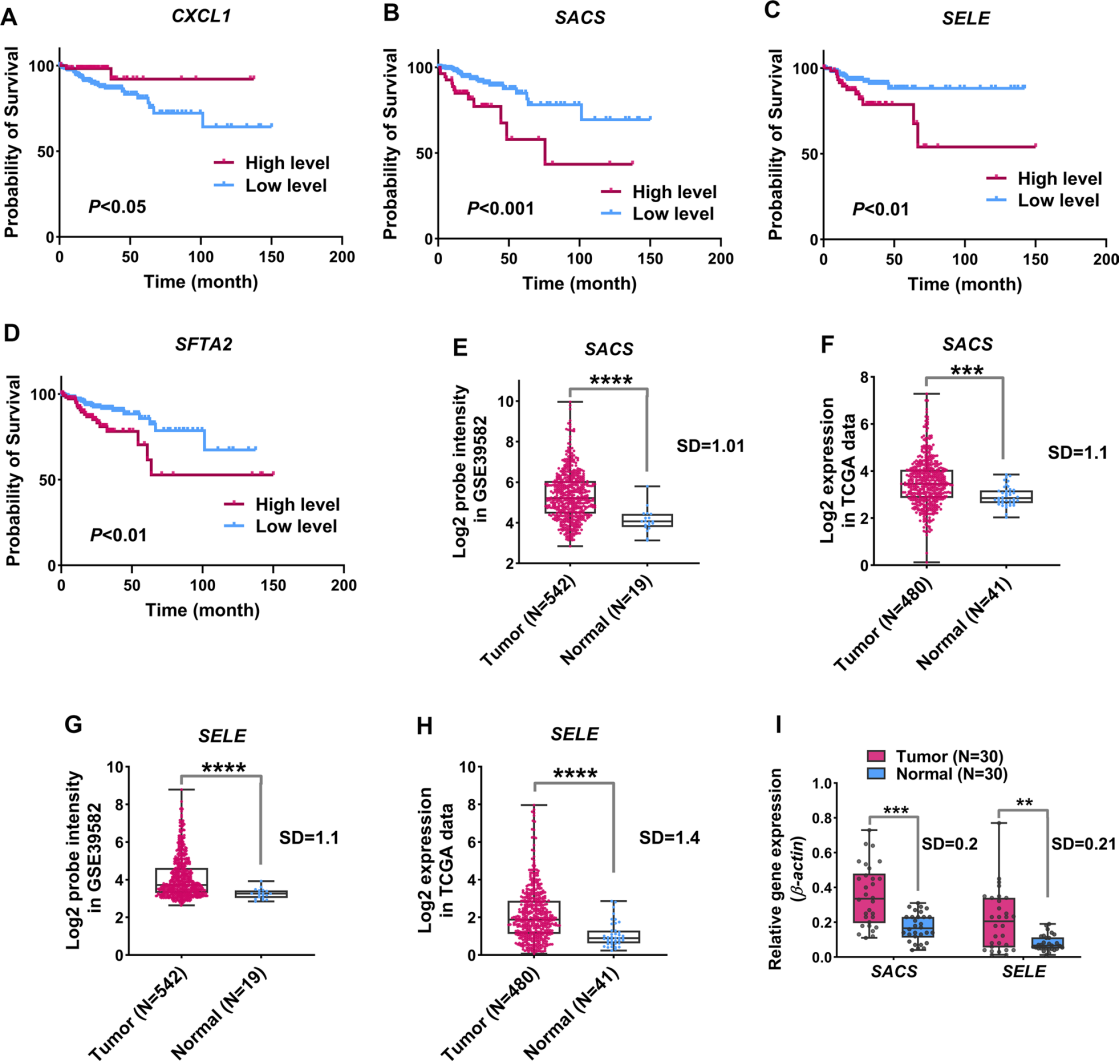
High heterogeneity of expression of *SELE* and *SACS* among CRC samples and their increased expression compared to normal samples. **A**–**D** Kaplan–Meier diagram for the expression level of *CXCL1*, *SFTA2*, *SELE,* and *SACS* and their relationship with patient survival based on TCGA data. The expression median of each gene was used as a cut off to divide the samples into groups with high and low expression. **E**–**H** The expression level of *SELE* and *SACS* genes in the two cohorts used in this study and their scattering level. **I** The expression level and dispersion of the expression of *SELE* and *SACS* genes in 30 colorectal cancer samples compared to normal samples by RT-qPCR

The expression of the remaining 83 genes with HHE and the survival of CRC patients were evaluated using TCGA clinical data. After initial preprocessing of clinical data from 480 cancer samples, 367 samples satisfied the materials and methods section's requirements and were used for survival analyses. According to the findings of Cox regression analysis, 12 genes were related with patient survival out of 83 identified genes with HHE, as shown in Table 1 and Fig. 3B (LogRank < 0.01). The findings of multivariate Cox regression revealed that the *CXCL1* was linked with good patient survival, whereas the *SFTA2*, *SELE*, and *SACS* expression were associated with poor patient survival, independently of other clinical characteristics (Table 1, LogRank < 0.01). In addition, the risk score for each patient was calculated based on the expression of these four genes and the results showed that these genes were strongly able to predict the survival of CRC patients (Fig. 3C and D, LogRank < 0.01). Moreover, the expression of genes in high-risk and low-risk groups in terms of specificity and sensitivity were analyzed by receiver operating characteristic (ROC) curve. The results showed that other than *SFTA2*, other genes could be highly sensitive in predicting groups from each other and serve as a good biomarker for identifying high-risk groups from low-risk (Fig. 2E, P < 0.001). For further confirmation, expression of *CXCL1*, *SFTA2, SELE*, and *SACS* were analyzed based on expression data and their classification by median criterion. The findings revealed that the expression of these four genes was linked to patient prognosis, confirming the findings of the previous phases (Fig. 4A–D, P < 0.05). These results suggest that genes with HHE can play a role in patient’s prognosis and could be used as markers to identify different survival rates of patients.

**Table 1:** Cox regression results for candidate genes with HHE based on TCGA clinical data

	Univariate			Multivariate		
	HR	*P* value	95% CI	HR	*P* value	95% CI
Age (60 > vs. < 60)	1.8	0.12	0.92–3.39			
Gender (Female vs. male)	1.4	0.23	0.72–2.63			
Pathological stage (P1-P2 vs. P3-P4)	11.9	2.4E-09	4.2–33.6	11.3	**0.0008**	3.37–18.23
TNM T stage (T1-T2 vs. T3-T4)	9.3	0.007	1.27–21.32	1.76	0.59	0.21–14.67
BLACAT1 expression (High vs. low)	1.37	0.03	1.01–1.86	1.19	0.34	0.82–1.73
SFTA2 expression (High vs. low)	1.43	0.007	1.09–1.86	1.53	**0.01**	1.1–2.13
S100A2 expression (High vs. low)	1.35	0.03	1.03–1.77	1.36	0.09	0.94–1.97
BGN expression (High vs. low)	1.39	0.03	1.02–1.9	0.69	0.38	0.3–1.58
SERPINE1 expression (High vs. low)	1.52	0.002	1.16–2.1	1.09	0.73	0.64–1.87
COMP expression (High vs. low)	1.37	0.02	1.03–1.83	0.80	0.54	0.4–1.64
THBS2 expression (High vs. low)	1.38	0.04	1–1.89	0.65	0.37	0.25–1.66
SELE expression (High vs. low)	1.33	0.01	1.04–1.69	2.1	**0.001**	1.32–3.48
SACS expression (High vs. low)	1.39	0.02	1.03–1.87	1.6	**0.01**	1.11–2.29
PBK expression (High vs. low)	0.67	0.006	0.52–0.89	0.76	0.24	0.49–1.19
SLC38A5 expression (High vs. low)	0.72	0.01	0.53–0.93	0.75	0.11	0.53–1.06
CXCL1 expression (High vs. low)	0.73	0.03	0.54–1	0.58	**0.007**	0.40–0.87

The expression matrix of genes in the Z-score scale was used to analyze the relationship between gene expression and survival

### Observing a lot of variability at the *SELE* and *SACS* gene expression levels in CRC samples

In order to confirm the obtained results for HHE genes, the expression level of *SELE* and *SACS* in CRC samples was evaluated and compared with normal samples. The results of the previous stage revealed that the expression levels of these two genes in cancer samples are highly heterogeneous in TCGA and GSE39582 data (Fig. 4E–H, SD > 1). The expression of *SELE* and *SACS* increased considerably in cancer samples compared to normal samples, according to the results of RT-qPCR data (Fig. 4I, P < 0.01). Moreover, the expression of these two genes had high heterogeneous behavior among CRC samples (Fig. 4I, SD > 0.1), which this outcome confirmed the previously obtained results.

### Raising drug resistance due to increasing expression of HHE genes

**Fig. 5:**
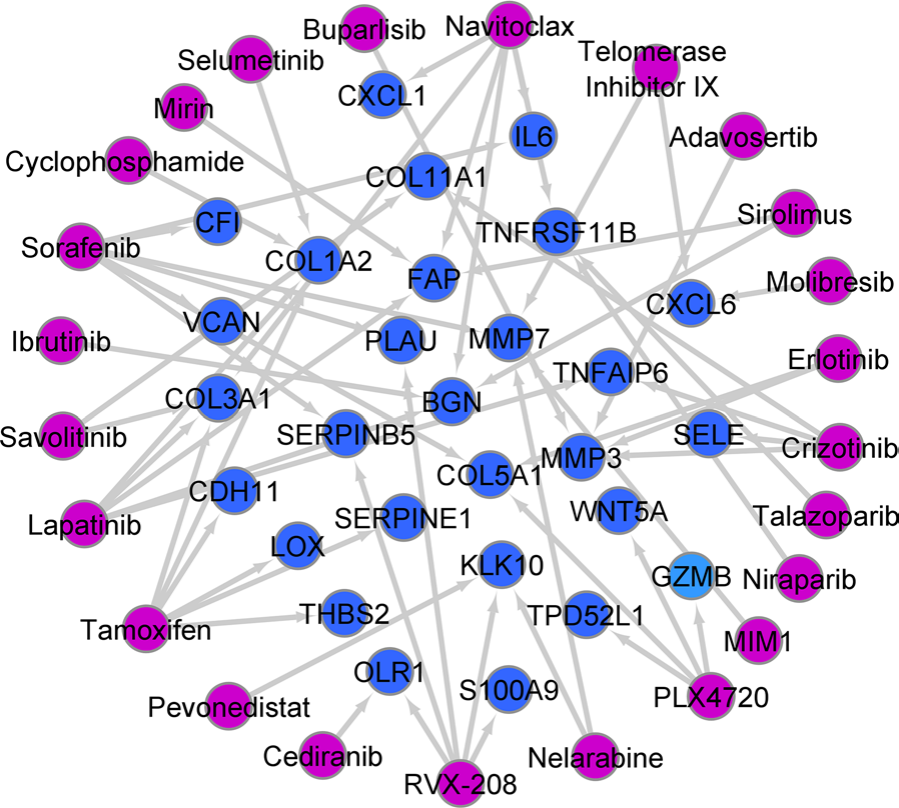
Correlating the expression of candidate genes with resistance to conventional chemotherapy drugs. Correlating the expression of 83 genes with HHE and IC50 of different drugs based on CCLE and GDSC data. Pearson correlation test was performed between the expression of candidate genes and IC50 of each drug in different cancer cell lines. A correlation coefficient greater than 0.2 and an FDR < 0.01 was considered to plot the relationship between the expression of each gene and each drug

Because variable therapeutic responses to medicines and the development of drug resistance are one of the most significant therapeutic problems for CRC, the impact of the expression of 83 genes classified as HHE linked to drug resistance was assessed. To assess the relationship between the expression of HHE genes with drug resistance, CCLE and GDSC data were used. Our findings revealed that a substantial number of potential genes were linked to chemotherapeutic drug resistance. As shown in Fig. 5, Sorafenib, Lapatinib, Navitoclax, Tamoxifen, and RVX-208 were associated with a large number of HHE genes (Fig. 5, FDR < 0.01). *SELE*, which previous results showed to be associated with poor prognosis, was associated with resistance to Crizotinib (Fig. 5, FDR < 0.01). Furthermore, the levels of BGN, MMP7, FAP, KLK10, and TNFRSE11B genes were linked to resistance to a variety of common chemotherapeutic treatments (Fig. 5, FDR < 0.01). These results suggest that many of the identified genes as HHE genes in CRC can participate with drug resistance. Therefore, the mentioned genes could be suitable biomarkers in choosing the appropriate drug and different therapeutic responses.

## Discussion

Personalized medicine and targeted therapy based on the molecular characteristics of each individual have been able to increase the cost of medical care and recovery of patients [17]. Moreover, extensive studies have shown that most cancers, especially CRC, have a high degree of heterogeneity at the molecular level. This feature can cause different therapeutic responses and different prognosis for patients with the same symptoms [18]. Therefore, many studies have been performed to identify common features of cancer cells and their different molecular features in order to improve treatment and perceive the mechanisms of CRC. In fact, identifying different molecular features in cancer samples could be a good therapeutic goal for personal medicine, and for patient’s state improvement.

Heterogeneity of expression level is a common feature in many cancers like CRC. Many studies have shown that tumor specimens with the same clinical symptoms have a range of differences in many gene levels, and this issue is associated with pathogenic complexities and difficult patient perception [19]. The genes that had high levels of heterogeneity in CRC samples investigated in this study. Two cohort studies were employed for this, and genes with significant variability of expression level were examined. The findings revealed that 132 genes were substantially up-regulated in CRC and that these genes were linked to a wide range of expression variations between tumor samples. The enrichment results of the obtained genes showed that many of these genes were associated with pathways including inflammation, angiogenesis, hypoxia, metastasis, and pathways associated with mutations. Some relevant literature indicated that inflammatory pathways are widely activated in cancer samples and are associated with proliferation, drug resistance, metastasis, and patient survival [20]. In addition, breast cancer has been shown to have extensive molecular heterogeneity associated with inflammatory genes [21]. Furthermore, some articles have reported that pathways associated with hypoxia are abundantly activated in tumor specimens although this pathway is associated with high heterogeneity [22]. These findings imply that these pathways might be good candidates for detecting changes across specimens and as CRC treatment targets. The results of this investigation revealed that *CXCL1*, *SFTA2*, *SELE*, and *SACS* expressions were considerably elevated in CRC and were linked to HHE. In addition, the expression level of these genes was associated with patient’s survival, and our results showed that their expression could significantly predict the risk of patient mortality. Furthermore, as compared to normal samples, tumor samples had higher levels of *SELE* and *SACS* expression, and tumor samples had a wide range of expression for these genes. Anne et al. showed that although the expression of *CXCL1* increases in tumor samples and the decrease in its expression could reduce cell proliferation, the increase in its expression is associated with a heterogeneity of the samples. In addition, *CXCL1* expression was only associated with a poor prognosis in stage IV samples. Our results displayed that in general, *CXCL1* in all samples was associated with a good prognosis of patients and this discrepancy seems to be due to not considering the high number of samples and classification of samples based on the stage in the relevant study [23]. In addition, studies have reported that high levels of *SELE* expression are associated with poor prognosis in patients with CRC [24]. These findings suggest that the expression of *CXCL1*, *SFTA2*, *SELE*, and *SACS* genes in CRC is highly heterogeneous and may be an important reason for the different prognoses of patients.

MMP7 expression levels are demonstrated to influence Sorafenib sensitivity [25]. Our results also showed that the expression level of *MMP7* in tumor samples was highly heterogeneous, and its expression level associated with resistance to Sorafenib. The findings of this study also revealed that *FAP* expression was higher in CRC samples and that this higher expression was linked to Navitoclax resistance. Studies have reported that *FAP* expression could regulate resistance to various drugs, including Navitoclax [26]. These data suggested that the number of genes identified as having a high degree of heterogeneity in CRC could impact chemotherapeutic treatment resistance. Therefore, identifying them can help to choose the suitable drug. However, the obtained results show a correlation, and this issue requires epidemiological and in vitro studies. Finally, identified genes as HHE, which found that while the expression of these genes in CRC increased in some samples, their expression reduced in another sample when compared to normal. It's also possible that varied treatment responses and prognoses for CRC patients are caused by the expression of identified genes. Therefore, the 83 genes reported in this study could be good candidates for personalized medicine and targeted therapy in CRC.

## Conclusion

The findings of this investigation revealed that the expression of 83 genes in CRC tissues might have a wide range of expression. In addition, our results established that *CXCL1*, *SFTA2*, *SELE*, and *SACS* had high heterogeneity of expression and were associated with patient prognosis. Furthermore, the expression of some identified genes such as *MMP7*, *FAP*, *KLK10*, and *TNFRSE11B* were correlated with several common chemotherapy drugs. The expression level of these genes could show the difference between CRC samples and be useful in targeted improvement and treatment of patients.

## Supplementary Information

**Additional file 1: Table S1.** List of 132 identified genes with high heterogeneity of expression.

**Additional file 2: Table S2*.*** Relationship between expression of HHE genes and clinical and genomic features.

## Data Availability

Supporting and raw data are available upon a reasonable request to the corresponding author.

## Abbreviations

CRC: Colorectal cancer
HHE: High heterogeneity of expression
TCGA: The cancer genome atlas
MSI: Microsatellite instability
MSS: Microsatellite stable
CCLE: Cancer cell line encyclopedia
GDSC: Genomics of drug sensitivity in cancer
ROC: Receiver operating characteristic
