# Supplementary material for: Identification of genes with high heterogeneity of expression as a predictor of different prognosis and therapeutic responses in colorectal cancer: a challenge and a strategy
Source: Cancer Cell Int. 2022 Sep 5;22:276. doi: 10.1186/s12935-022-02694-9 (PMC9446546; doi:10.1186/s12935-022-02694-9)
Supplement: Supplementary file 2 — Additional file 2: Table S2. Relationship between expression of HHE genes and clinical and genomic features. [file 12935_2022_2694_MOESM2_ESM.docx]

**Table S2:** Relationship between expression of HHE genes and clinical and genomic features.

| **Gene names** | **Dependence on clinical and genomic properties** |
| --- | --- |
| RNF43,DPEP1, APCDD1, CEL, ASCL2 | APC mutation |
| KCNN4, TCN1, CA9, LEMD1 | KRAS mutation |
| COL9A3, KRT23 | Stage IV |
| DUSP4, ZIC2, CD55, CLDN2 | Cecum site |
| GPR143, CEL, SLC35D3, PMEPA1, ERP27, KRT23, RNF43, ASCL2, APCDD1,DPEP1, MME, AGT, LAPTM4B, DACH1, SFRP4, UCA1 | Sigmoid site |
| DUSP4, CXCL10, RPL22L1, ZIC2, CD55, CLEC5A, FSCN1, HSPA4L, MMP12, TREM1, CXCL11, MMP1, PRRX1, CXCL8, SPP1, TMPRSS3, REG1A, LY6E, REG1B, HS6ST2, IGF2BP3, RNF43, KRT23, ASCL2, DPEP1, PMEPA1, GPR143, CEL, APCDD1, LAPTM4B, DACH1, ERP27, AZGP1, SLC35D3, RASSF10, AGT, APOLD1, FOXQ1, EDAR, COL9A3, UCA1, LGR5 | MSI and MSS state |
